# Early Results of the A.L.P.S. Proximal Humerus Locking Plate

**DOI:** 10.2174/1874325001812010053

**Published:** 2018-02-28

**Authors:** Miltiadis Argyropoulos, Matthew Kent

**Affiliations:** Department of Trauma & Orthopaedics, Royal Liverpool & Broadgreen University Hospital Trust, Appointment of Registrar Trauma & Orthopaedics, Prescot St, Liverpool L7 8XP, UK

**Keywords:** Proximal humerus, fracture, A.L.P.S., ALPS, locking plate, trauma, fixation

## Abstract

**Background:**

Open reduction internal fixation of displaced 3 and 4-part proximal humerus fractures is an effective treatment modality particularly for the younger age group, but it is not without complications. Fixed angled locking screw plates are the mainstay of fixation. The A.L.P.S. Proximal Humerus Plating System is a new implant that has smooth locking pegs in the place of humeral head screws to help reduce glenoid damage in the event of cut-out and is designed to sit low on the humerus in order to reduce the risk of subacromial impingement.

**Methods:**

Retrospective analysis of 15 consecutive patients who sustained closed displaced 3-part or 4-part fractures and had fixation surgery using the A.L.P.S. plate. Outcome measures were the time to radiographic and clinical union, Oxford Shoulder Score, quick DASH score and complications.

**Results:**

Average follow-up was 31.9 weeks. Union was achieved in 100% of patients with a mean time to union of 15.1 weeks. In terms of function, mean OSS was 33.6 and mean quick DASH was 32.5. There were no instances of AVN.

**Conclusion:**

Our preliminary results of 15 patients followed up for a mean of 31.9 weeks show equitable union rates and time to union as well as functional scores compared to other available plating systems. This is the first study to report on this implant to date.

## 1 INTRODUCTION

Proximal humerus fractures comprise 4-5% of the incidence of all fractures [1] and are the most common fractures of the humerus. Approximately 80% of proximal humerus fractures are stable and minimally displaced, and can be treated non-operatively [2, 3]. Operative options for the management of displaced 3-part and 4-part fractures include open reduction internal fixation (ORIF) and arthroplasty. ORIF aims to preserve bone stock, restore anatomy and prevent glenoid erosion. Anatomical proximal humerus locking plates have been used extensively, a well established and reported implant being the Proximal Humeral Internal Locking System P.H.I.L.O.S. (Synthes, Solothurn Switzerland) [4-6]. This is a fixed angle device which is thought to provide rotational stability particularly in osteoporotic bones [7] with a further goal being early mobilization. Despite good overall results, avascular necrosis (AVN) following ORIF stands at approximately 16% with a further complication being screw cut-out subsequent to collapse, reported at 14% [8] and subacromial impingement at 6% [9].

We are reporting the early results of a new implant, the A.L.P.S. Proximal Humerus Plating System (Zimmer Biomet, Warsaw, Indiana, USA) which aims to address certain complications with the following design features. Smooth blunt ended pegs are used instead of screws in the head to reduce injury and irritation in the event of intra-articular penetration due to collapse. The plate is designed to sit low on the humeral neck with the aim to reduce the likelihood of subacromial impingement [10], much like its predecessor the S3 [11] (Zimmer Biomet, Warsaw, Indiana, USA). A high option exists for situations where a better proximal hold is required providing 2 additional screw options more proximally. Further holes are available for K-wires and sutures. The plate is designed to fix the humerus at a 135 degree neck-shaft angle. Risk of varus collapse is minimized by creating an internal subchondral support system of diverging and converging locking screws and a medial calcar screw. Our aim was to report the early results of this implant as one of the first centres to adopt it.

## 2 METHODS

The first 15 consecutive patients receiving the A.L.P.S. plate starting from when we introduced this implant in our unit between June 2016 and January 2017 were followed up to assess their outcomes. The inclusion criteria were patients sustaining closed displaced 3-part and 4-part fractures who were fit for surgery, as shown in Fig. (**1**). The decision to perform ORIF was dependent on surgeon preference. Lead surgeons were either specialist upper limb consultants or fellows under consultant supervision. The surgery was performed via a deltopectoral approach Fig. (**2**). The fracture was reduced Fig. (**3**) and heavy sutures used to control the rotator cuff and associated fragments Fig. (**4**). A high Fig. (**5**) or a low plate was applied to the proximal humerus following reduction depending on surgeon choice. The greater tuberosity was repaired with additional sutures and secured to the plate. Pegs were used to immobilize the head fragments. Bone void filler Cerament (Bonesupport AB, Lund, Sweden) was used at the surgeons’ discretion. The entire procedure was performed under radiographic control. Fig. (**6**) shows final intraoperative radiographic appearance of high A.L.P.S. plate in situ with smooth pegs and Cerament. Post-operatively all patients received physiotherapy. Outcome measures were the time to radiographic and clinical union, Oxford Shoulder Score, quick DASH and complications were also recorded. Union was defined as the time point when both radiographic and clinical union had been achieved.

## 3 RESULTS

Out of 15 patients, 11 were female and 9 out of the 15 were right sided injuries. Mean age at the time of surgery was 56.5 (range 36-68). The mechanism of injury was low energy mechanical fall in all cases. There were 10 patients with 3-part fractures and 5 with 4-part fractures. There were 3 smokers and 1 non-insulin-dependent diabetic. Average time to surgery was 9.3 days (2-27) median was 8 days. High plates were used in 13 patients and bone void filler was used in 9.

Average follow-up was 31.9 weeks (range 14 - 45). There was 1 patient lost to follow-up following discharge from our clinic due to death from unrelated cause. Union was achieved in 100% of patients. Mean time to union was 15.1 weeks and ranged from 7 to 23 weeks. Mean OSS was 33.6 (range 23 - 46) and mean quick DASH was 32.5 (range 2.3 - 56.8). There was 1 deep wound infection which required formal washout and 1 superficial infection which settled with oral antibiotics alone. There was 1 patient who was symptomatic due to glenohumeral joint impingement of the pegs, however this was not due to collapse, but due to incorrect insertion at the time of the procedure. This patient is due to undergo removal of metalwork. There was 1 patient who was found to have glenohumeral impingement due to peg protrusion following osteoporotic collapse, however an operation to remove metalwork was declined due to acceptable symptoms and function. A further patient is due to have a capsular release and MUA for stiffness and the plate will also be removed however there was no intra-articular penetration of the pegs. There was 1 postoperative HDU admission due to chest infection. There were no instances of AVN during our follow-up period.

## 4 DISCUSSION

ORIF of displaced 3 and 4 part proximal humerus fractures is an effective management option particularly in the younger patients where it is important to maximise function and preserve bone stock. Subacromial impingement is a reported complication of ORIF as the proximally fixed plate impinges under the acromion during shoulder abduction. The A.L.P.S. plate is a low profile locking plate which comes in two versions, a low and a high sitting plate. Our study suggests that although the low plate did not result in subacromial impingement in any of our patients, a high plate was needed in the majority of occasions in order to allow for satisfactory reduction and fixation. This implant appears to have equitable functional scores compared to the PHILOS plate [12].

There were two instances where there was intra-articular penetration of the pegs. One patient opted for removal and the other declined it, suggesting that smooth pegs may offer an advantage over screws in these situations.

## CONCLUSION

This study shows that the A.L.P.S. plate is a safe implant with equitable union rates. A weakness of this study is the short follow-up time. This may underestimate the functional and symptomatic efficacy of the implant. Furthermore, long-term complications such as AVN may go unreported. Further research with greater numbers and follow-up is required to elucidate whether its unique design features have the intended effects on metalwork irritation and impingement as well as to assess the learning curve associated with using this implant and to compare it with other proximal humerus locking plates available on the market.

## Figures and Tables

**Fig. (1):**
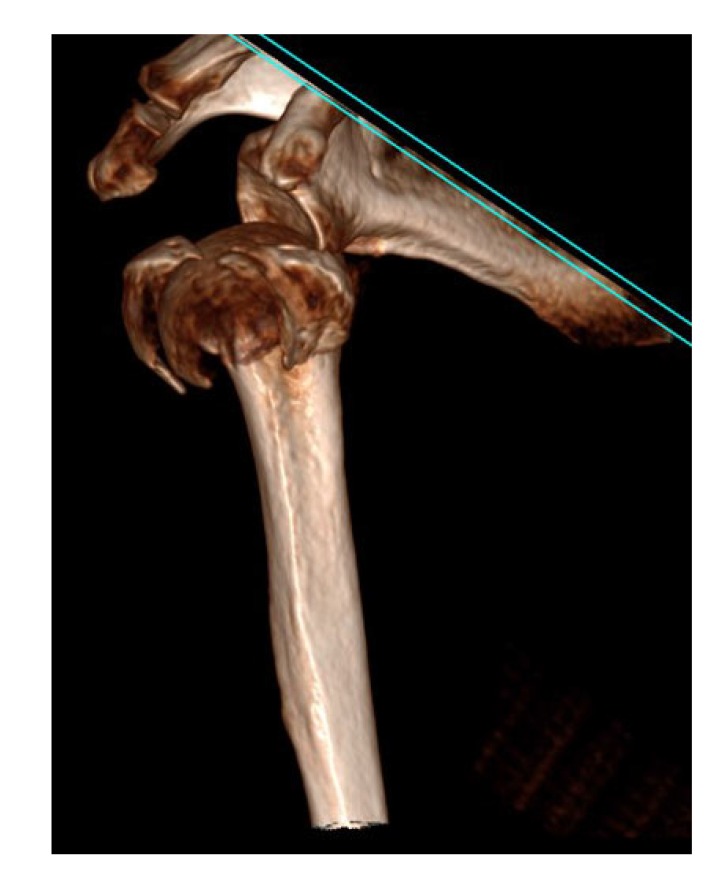
3D reconstruction of CT scan showing 4-part proximal humerus fracture.

**Fig. (2):**
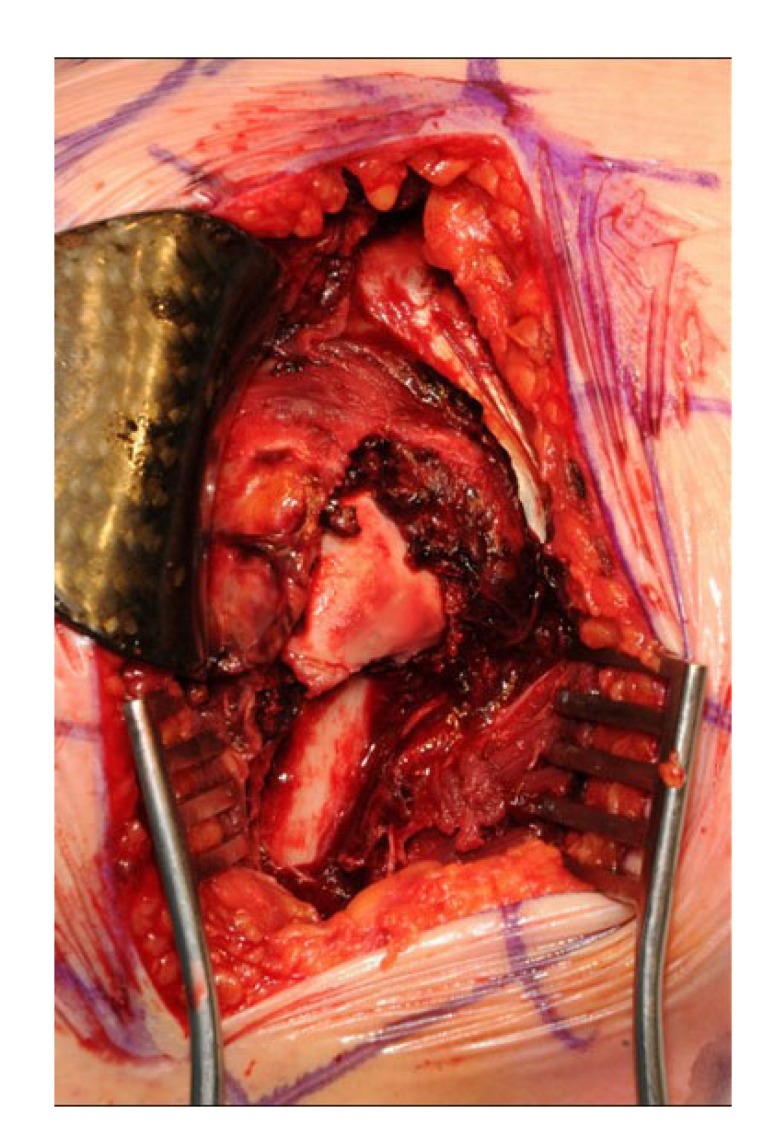
Fracture appearance following deltopectoral approach.

**Fig. (3):**
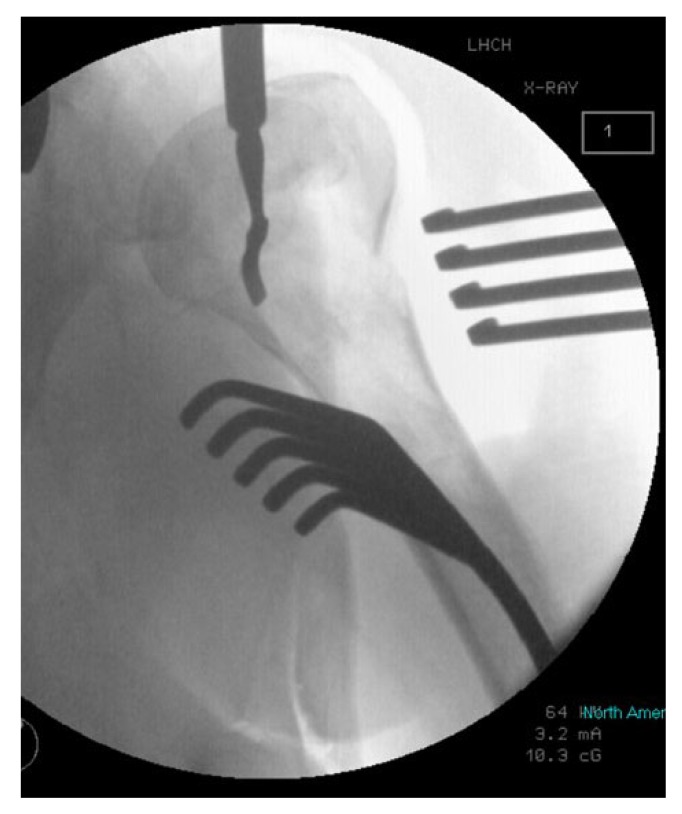
Intra-operative radiograph depicting fracture reduction.

**Fig. (4):**
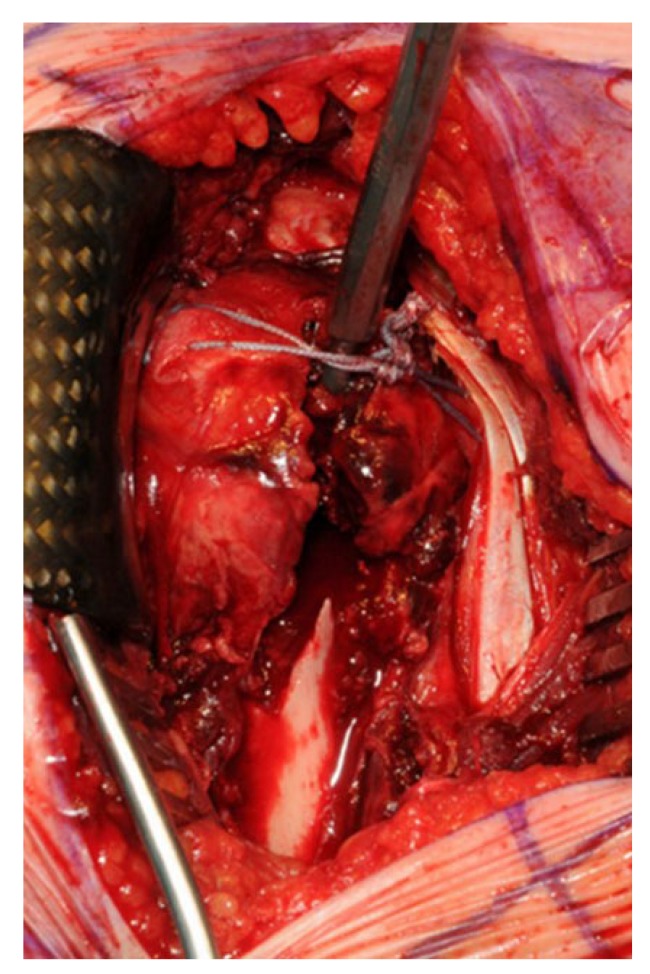
Greater tuberosity reduced using heavy sutures.

**Fig. (5):**
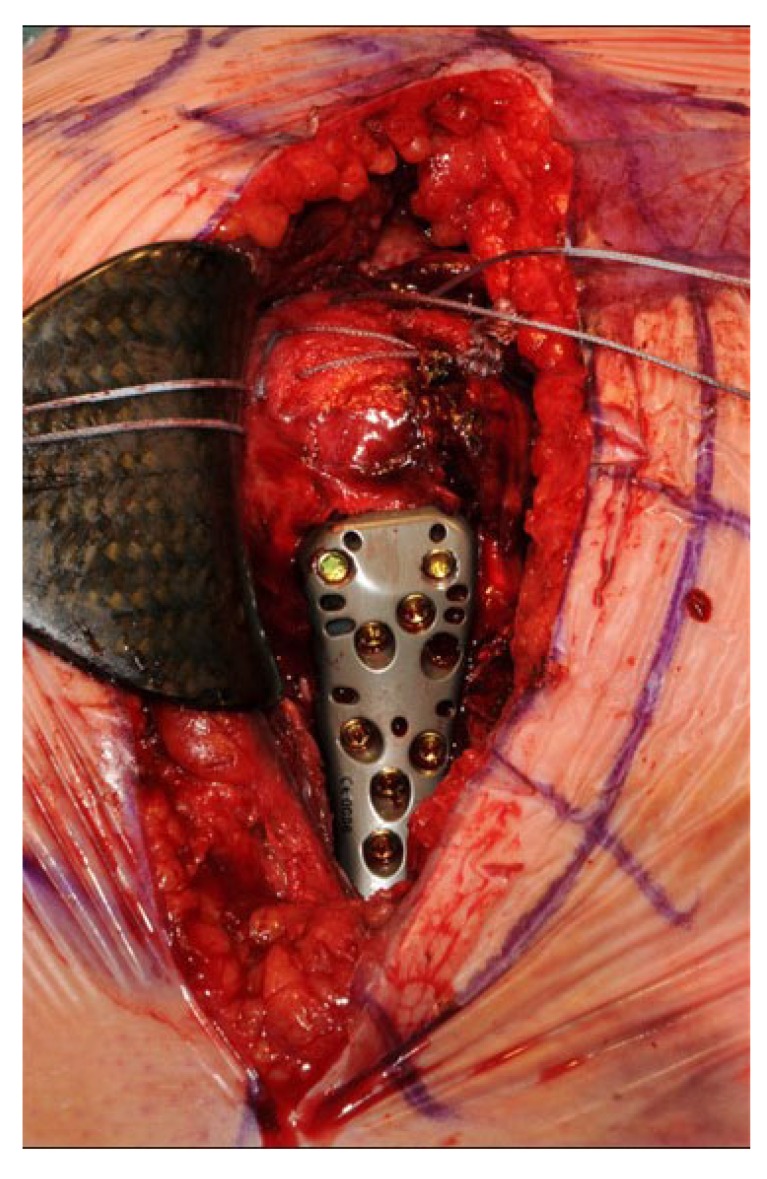
High A.L.P.S. plate *in situ*.

**Fig. (6):**
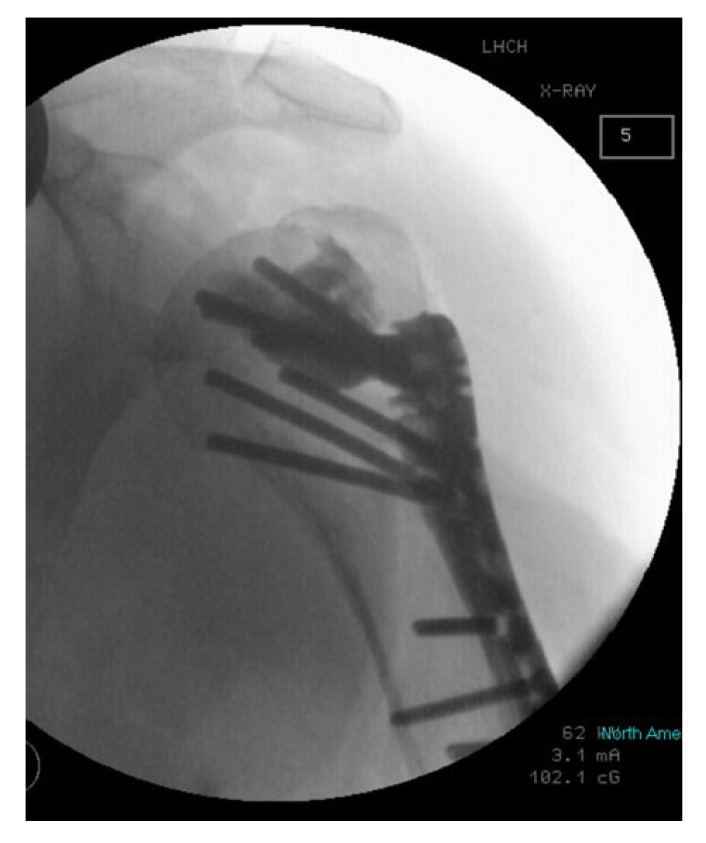
Final intra-operative radiograph showing A.L.P.S. plate with smooth pegs and Cerement.

## LIST OF ABBREVIATIONS

A.L.P.S.: = Anatomical Locking Plate System
AVN: = Avascular Necrosis
DASH: = Disabilities of the Arm, Shoulder and Hand
HDU: = High Dependency Unit
MUA: = Manipulation Under Anaesthetic
ORIF: = Open Reduction Internal Fixation
P.H.I.L.O.S.: = Proximal Humeral Internal Locking System
